# The Chemical Compositions of Essential Oils Derived from *Cryptocarya alba* and *Laurelia sempervirens* Possess Antioxidant, Antibacterial and Antitumoral Activity Potential

**DOI:** 10.3390/molecules25235600

**Published:** 2020-11-28

**Authors:** Jorge Touma, Myriam Navarro, Betsabet Sepúlveda, Alequis Pavon, Gino Corsini, Katia Fernández, Claudia Quezada, Angelo Torres, María José Larrazabal-Fuentes, Adrian Paredes, Ivan Neira, Matías Ferrando, Flavia Bruna, Alejandro Venegas, Jessica Bravo

**Affiliations:** 1 Facultad de Medicina, Centro de Investigación Biomédica, Universidad Diego Portales, Ejército 141, Santiago 837007, Chile; jorge.touma@mail.udp.cl (J.T.); Myriam.navarro@mail.udp.cl (M.N.); Katia.fernandez@udp.cl (K.F.); alejandro.venegas@mail.udp.cl (A.V.); 2 Facultad de Ciencias Químicas y Farmacéuticas, Universidad de Chile, Santos Dumont 964, Santiago 8880494, Chile; bsepulveda@ciq.uchile.cl; 3 Instituto de Ciencias Biomédicas, Facultad de Ciencias de la Salud, Universidad Autónoma de Chile, Llano Subercaseaux 2801, Santiago 8910060, Chile; apavon@autonoma.cl (A.P.); gcorsini@autonoma.cl (G.C.); 4 Laboratorio de Patología Molecular, Instituto de Bioquímica y Microbiología, Facultad de Ciencias, Universidad Austral de Chile, Valdivia 5110566, Chile; claudiaquezada@uach.cl (C.Q.); angelo.uach.2017@gmail.com (A.T.); 5 Laboratorio Química Biológica, Departamento de Ciencias de los Alimentos y Nutrición, FACSA, Universidad de Antofagasta, Angamos 601, Antofagasta 1270300, Chile; maria.larrazabal@uantof.cl (M.J.L.-F.); adrian.paredes@uantof.cl (A.P.); ivan.neira@uantof.cl (I.N.); 6 Laboratorio de Hormonas y Biología del Cáncer, Instituto de Medicina y Biología Experimental de Cuyo (IMBECU), CONICET CCT-Mendoza UNcuyo 5500, Argentina; MFerrando@gmail.com (M.F.); flabruna@gmail.com (F.B.)

**Keywords:** essential oil, *Cryptocarya alba*, *Laurelia sempervirens*, chemical composition, antioxidant, cytoxicity, toxicity, antimicrobial

## Abstract

*Cryptocarya alba* (Peumo; CA) and *Laurelia sempervirens* (Laurel; LS) are herbs native to the Chilean highlands and have historically been used for medicinal purposes by the Huilliches people. In this work, the essential oils were extracted using hydrodistillation in Clevenger apparatus and analyzed by GC-MS to determine their composition. The antioxidant capacity (AC) was evaluated in vitro. The cytotoxicity was determined using cell line cultures both non tumoral and tumoral. The toxicity was determined using the nematode *Caenorhabditis elegans*. The antimicrobial activity was evaluated against 52 bacteria using the agar disc diffusion method and the minimum inhibitory concentrations (MICs) were determined. The principal compounds found in *C. alba* essential oil (CA_EO) were α-terpineol (24.96%) and eucalyptol (21.63%) and were isazafrol (91.9%) in *L. sempervirens* essential oil (LS_EO). Both EOs showed antioxidant capacity in vitro. Both EO showed antibacterial activity against bacteria using. LS_EO showed more inhibitory effect on these cell lines respect to CA_EO. Both EOs showed toxicity against the nematode *C.* *elegans* at 3.12–50 mg/mL. The essential oils of CA and LS have an important bioactive potential in their antioxidant, antibacterial and cytotoxicity activity. Both essential oils could possibly be used in the field of natural medicine, natural food preservation, cosmetics, sanitation and plaguicides among others.

## 1. Introduction

*Cryptocarya alba* and *Laurelia sempervirens* essential oil (EO) plant species used by the Huilliche people of Chile for wound treatment and associated infections, were evaluated against bacterial and fungal human pathogens [1]. Essential oils exhibit a wide range of bioactivities. The antimicrobial activity has played a key role in the utilization of these EOs for the treatment of various human diseases. 

Cancer is an important cause of morbidity and mortality worldwide [2]. The increase of the incidence indicates that 1:8 men and 1:10 women will develop this multifactorial disease in a lifetime [2]. Due to the aforementioned, the research for alternative and complementary treatments of cancerous diseases still motivates the search for new antitumoral agents [3]. The EOs obtained from diverse plants are able to increase the efficacy of commonly used chemotherapy drugs, having also shown pro-immune functions when administered to cancer patients [4]. It has been reported that the EOs may interfere in various signaling pathways in cancerous cells and may exert anti-mutagenic, anti-proliferative, antioxidant and detoxifying effects [3]. The MCF-7 line is one of the most described cell lines in the literature for testing antitumor activity of natural bioactive products. The cytotoxic effects on MCF-7 cell line treated with EOs from plants of the genus *Cryptocarya* [5,6] and *Laurelia* [7] had previously been reported. 

In Chile, research on antimicrobial compounds obtained from native plants has yielded some positive results in the antimicrobial activity [1]. Moreover, Bittner [8] has evaluated the fungistatic activity of EO extracted from CA_EO and LS_EO against fungi such as *Rhizoctonia solani* Kühn (Donk) and *Fusarium oxysporum* Schltdl. Additionally, Avello [9] evaluated the antifungal activity of EOs extracted from canelo, queule, bailahuén and culen against other phytopathogenic fungi, such as *Botritys cinerea*, *Fusarium oxysporum* and *Aspergillus niger*. Additionally, we have identified the major active compounds as well as evaluated the effects of the CA_EO against *N. ceranae* and its potential use for the control of nosemosis disease [10].

The aim of this work was to evaluate the chemical composition of the EOs extracted from *C. alba* and *L. sempervirens* and their antioxidant capacity, cytotoxicity activity and antibacterial potential.

## 2. Results

### 2.1. Composition

The sample compositions obtained by hydrodistillation using the Clevenger-type apparatus Table 1 indicated that the *C. alba* essential oil (CA_EO) contained 14 compounds, while the *L. sempervirens* essential oil (LS_EO) contained 6 compounds. The highest yielding compounds in CA were α-terpineol (24.96%), eucalyptol (21.63%), and β-phellandrene (14.84%) and the highest yielding compounds in LS were isazafrol (91.9%), limonene (5.3%), and *O*-cimene (1.3%).

### 2.2. Antioxidant Capacity

The antioxidant potential of CA and LS EOs was evaluated using the following complementary techniques: total Polyphenols contents, FRAP and radical scavenger DPPH and ABTS methods. The results are showed in Table 2. The EOs showed significant antioxidant activity with variability in the different methods used.

### 2.3. Antimicrobial Activity

**Figure 1:**
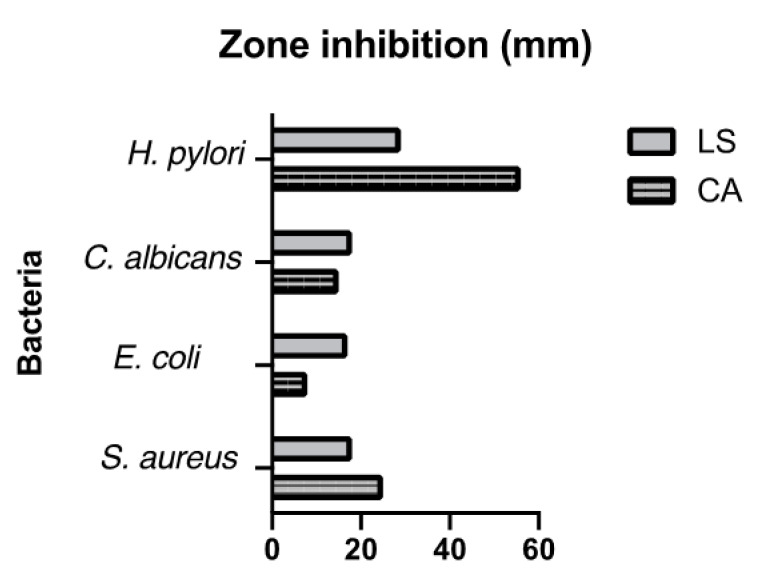
Zone of Inhibition (mm) resulting from CA (*Cryptocarya alba*) and LS (*Laurelia sempervirens*) EOs obtained by hydrodistillation in a Clevenger-type apparatus.

The antimicrobial activity of the CA_EO and LS_EO are shown in Table 3. The CA_EO was the most effective against *S. aureus, E. coli, C. albicans and H. pylori* with inhibition zones of 25, 8, and 15 mm (Figure 1), respectively and complete inhibition at the same concentration against *H. pylori*. The essential oil was shown to have antimicrobial activity against both Gram-positive and Gram-negative bacteria. Antimicrobial activity against *E. coli* was slightly less than that against *S. aureus* and *C. albicans* (*p* < 0.05) at the same concentration. The MICs of the CA_EO against the bacteria mentioned above were 19.0, 36.0, 31.0, and 30.0 μg/mL respectively, and LS_EO against the bacteria mentioned above were 64.0 μg/mL.

### 2.4. Cytotoxicity Activity

**Figure 2:**
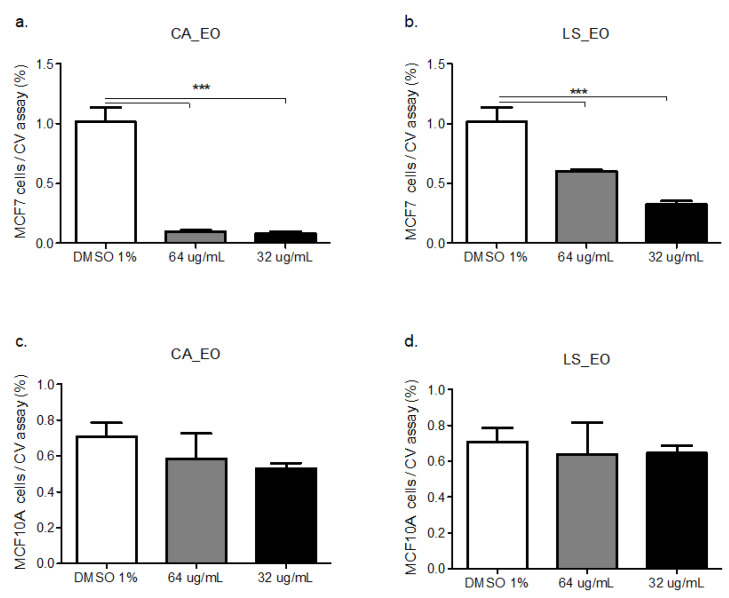
Dose-response curve of the human epithelial mammary tumor cell line MCF-7 (**a**,**b**) and epithelial mammary non-tumoral cell line MCF10A(**c**,**d**) treated with CA or LS EOs. The graph bar corresponds to the proliferation of MCF-7 treated with the EOs at different concentrations versus control (DMSO 1%) for 48 h, evaluated by CV assay at 570 nm. Three independent trials were performed, in triplicate, for each treatment and concentration. *** *p* < 0.001.

**Figure 3:**
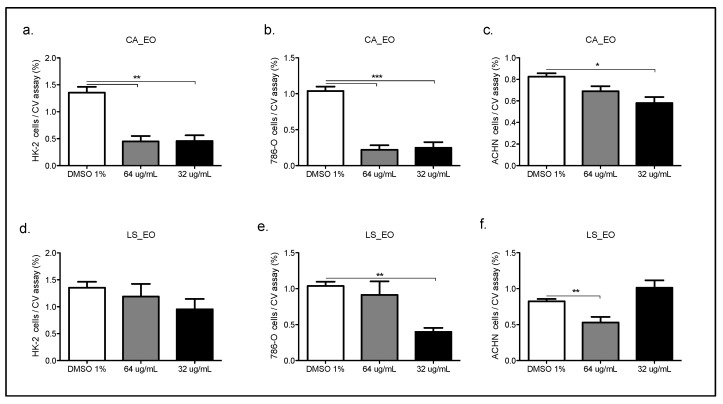
Dose-response curve of the human epithelial renal non-tumor cell line (HK2, **a**–**d**) and tumor cells line ((786-O, **b**–**e**) and (ACHN, **c**–**f**)), treated with CA or LS EOs. The graph bar corresponds to the proliferation of Hk2, 786-O and ACHN, treated with the EOs at different concentrations versus control (DMSO 1%) for 48 h, evaluated by CV assay at 570 nm. Three independent trials were performed, in triplicate, for each treatment and concentration. * *p* < 0.05, ** *p* < 0.005, *** *p* < 0.001.

**Figure 4:**
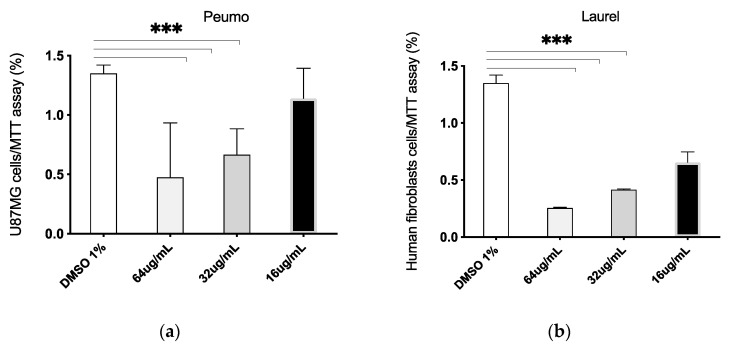
Dose-response curve of human glioblastoma cells tumor cell line U87MG treated with *Cryptocarya alba* (Peumo; CA); *Laurelia sempervirens* (Laurel; LS) EOs. The graph bar corresponds to the proliferation of U87GMtreated with the Eos (a. Peumo) and (b. Laurel), at different concentrations versus control (DMSO 1%) for 48 h, evaluated by MTT assay at 570 nm. Three independent trials were performed, in triplicate, for each treatment and concentration. *** *p* < 0.001.

**Figure 5:**
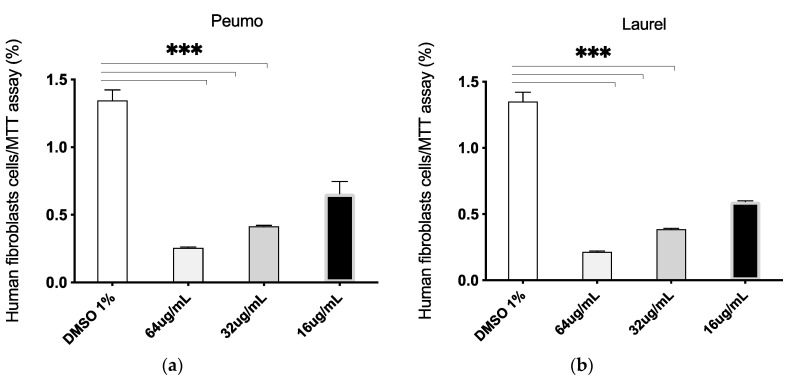
Dose-response curve of human fibroblasts cells treated with *Cryptocarya alba* (Peumo; CA); *Laurelia sempervirens* (Laurel; LS) EOs. The graph bar corresponds to the proliferation of fibroblasts cells treated with the Eos (**a**. Peumo) and (**b**. Laurel), at different concentrations versus control (DMSO 1%) for 48 h, evaluated by MTT assay at 570 nm. Three independent trials were performed, in triplicate, for each treatment and concentration. *** *p* < 0.001.

Both CA and LS EOs present selective inhibitory activity on epithelial tumor cells. Figure 2, Figure S3, Figure 3a–d, Figure 4 and Figure 5.

### 2.5. Toxicity

**Figure 6:**
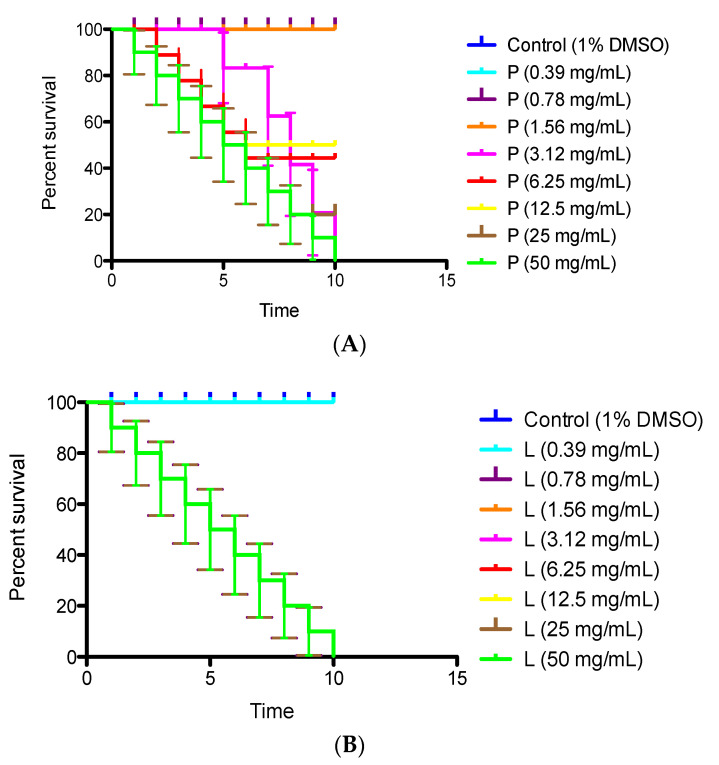
(**A**) Percent survival of P (Peumo, *C. alba*) and (**B**) L (Laurel, *L. sempervirens*) EOs against *C. elegans.*

CA_ EO showed low or no toxicity against *C. elegans* (Figure 6) while LS_ EO showed toxicity in all the concentrations evaluated, with 100% mortality, both at 24 and 48 h. CA_EO was toxic at 24 h at 50 with 100% mortality.

## 3. Discussion

The EO yields obtained using hydrodistillation by the HD process were 0.6% for CA and 1.08% for LS. These differ from those reported by other authors who extracted the EOs by hydrodistillation from the same herbs. For LS, the range was between 2.79% and 6.28% [8], while for CA and LS values were 0.76% and 0.7% respectively [9]. 

The sample compositions obtained by HD using the Clevenger-type apparatus (Table 1) indicated that the CA_EO contained 14 compounds, while the LS_EO contained 6 compounds. The highest yielding compounds in CA_EO were α-terpineol (24.96%), eucalyptol (21.63%), and β-phellandrene (14.84%) and the highest yielding compounds in LS were isazafrol (91.9%), limonene (5.3%), and O-cimene (1.3%). 

Zapata [11], reported that the LS_EO extracted by HD contained 16 compounds with safrole (82.4%) and limonene (7.76%) being the highest [11]. The CA_EO analysis CG-MS contained 6 compounds where 1-terpinen-4-ol (28.19%) and β-terpineno (23.08%) constituted the majority. The LS_EO contained 12 compounds where safrol (31.43%) and (1*S*)-α-pineno (27.91%) constituted the majority [9].

In the current study, the differences in the yield and composition of the CA and LS_EOs compared to those in the literature, could be due to both the extraction method and the collection time [12]. The chemical composition of the oils affects their bioactivity [13], even though their bioactivity should not be attributed to a particular compound [14]. Biological effects are the result of a synergism of all molecules contained in an EO even if it is possible that the activity of the main component is modulated by other minor molecules. The activity of the isolated constituents, however, is also notable [15]. Previously we demonstrated the CA_EO antifungal activity against *Nosema ceranae* [10]. Some of the compounds found in this work such as eucalyptol, (one of the major compounds in CA_EO) has been reported in other EOs included *Acantholippia deserticola* and demonstrated inhibition of *E. coli* [16]. Eucalyptol was ascertained in *P. boldus* and *L. philippiana* EO antifungal activity against *Fusarium oxysporum* Schltdl [8]. 

The total phenol content in the EOs was determined by the Folin-Ciocalteu spectrometric method. The amount of total content of phenols for both oils was moderate with a value of 63.7 ± 10.7 for EO_LS and significantly higher (*p* < 0.01) 163.6 ± 10.7 mg Gallic acid equivalent/g for CA_EO respectively. The FRAP method is based on the reduction of the Fe^3+^-TPTZ (iron-tripyridyltriazine) complex, to Fe^2+^-TPTZ, that is, it measures the capacity of the EO to reduce Fe^3+^ to Fe^2+^. For this assay the EO of LS was significantly more effective (*p* < 0.05) in the reduction of the complex Fe^3+^ with a value of 229.8 ± 11.1 mg Trolox equivalent/g EO, while the CA_EO presents values of 166.8 ± 27.9 mg Trolox equivalent/g EO respectively. Two complementary techniques were used to evaluate the potential free radical scavenger of EOs using the DPPH and ABTS radicals. When comparing the IC_50_ values, it was observed that both EOs exhibit moderate DPPH radical scavenging activity. The values for CA_ EO was 417.8 ± 5.8 µg/mL and 492.7 ± 11.1 µg/mL for LS_EO. Both oils scavenged the DPPH radical in a concentration-dependent manner. Neither of the two oils was as effective as the Trolox antioxidant (IC_50_ = 11.7 ± 2.1 µg/mL). The CA_EO showed a significantly (*p* < 0.01) greater ability to inhibit the ABTS radical compared to the LS_EO with IC_50_ values of 203.0 ± 12.8 µg/mL and 401.2 ± 8.7 µg/mL respectively (Table 2). Both EOs showed a significantly (*p* < 0.001) lower inhibitory activity than Trolox (IC_50_ = 35.6 ± 1.5 µg/mL). While no phenolic compounds were found and considering that CA_EO has a higher antioxidant potential than EO_LS, this could be attributed to compounds such as α-terpineol, γ-terpinene, β-phellandrene whose activity had been previously described by Amorati [17]. It could be inferred that the antioxidant activity of the *Eucalyptus globulus* oil is mainly due to the presence of its major compounds, namely 1,8-cineole (63.8%) [11]. Eucalyptol (1,8-cineole) showed various degrees of reducing power, radical scavenging and chelating capacity, in addition to its DNA-protective capacity [11]. Moreover, the high value of the reducing power indicated that the EOs components are able to act as electron donors and reduce the oxidized intermediate of lipid peroxidation so that they can act as primary and secondary antioxidants [13].

Total antioxidant activities of the EOs cannot be evaluated by any single method due to the complex nature of phytochemical composition [18]. Two or more methods should always be employed in order to evaluate the total antioxidative effects. In this sense, the CA_EO, showed a higher antioxidant activity, compared to that of LS_EO obtained with FRAP, DPPH or ABTS methods [19]. This may be due in part to the complexity of the chemical composition. The antioxidant activity shown by CA_EO can be explained by its greater composition in terpenes with their conjugated hexadiene structure (35% of its composition), in comparison to the content of 14% of compounds with the same carbon nucleus for the LS_EO. It has been shown that the compounds present in the CA_EO such as d-phellandrene (0.71%), β-phellandrene (14.84%) γ-terpinene (2.67%), α-terpinene (24.96%) and limonene (3.41%), act through a mechanism of autooxidation, trapping free radicals efficiently [12]. This higher antioxidant activity is also due to the fact that the CA_EO has in its composition hydroxylated terpenes such as β-eudesmol (1.49%), 4-terpineol (1.72%) and the second major component α-terpineol (24.29%). On the other hand, the LS_EO does not present hydroxylated terpenes and only 5.3% of limonene. Although phenolic aromatic compounds were not identified in both oils, there are reports of other EOs whose absence of this type of compound has presented a higher antioxidant capacity comparable to the synthetic antioxidant BHT [17,19,20].

The CA_EO was the most effective against *S. aureus, E. coli, C. albicans and H. pylori* with inhibition zones of 25, 8, and 15 mm (Figure 1) respectively. Additionally, the CA_EO showed a complete inhibition at the same concentration against *H. pylori* and showed antimicrobial activity against both Gram-positive and Gram-negative bacteria. At the same concentration antimicrobial activity against *E. coli* was slightly lower than that against *S. aureus* and *C. albicans* (*p* < 0.05). The MICs of the CA_EO against the bacteria mentioned above were 19.0, 36.0, 31.0, and 30.0 μg/mL respectively (Table 3). The MIC for LS_EO against the above-mentioned bacteria was 64.0 μg/mL. The chemical composition of CA_EO which is primarily composed of α-terpineol (27.96%), eucalyptol (21.63%), and β-phellandrene (14.84%). On the other hand, LS_EO which is composed by limonene (5.3%), showed strong antimicrobial activity against *S. aureus* and *H. pylori.*

The biological activity on the major monoterpenes reported for CA_EO components, has been previously described, as noted below. For instance, a study showed that a protective effect of standardized *Pistacia atlantica* EO against ethanol-induced gastric ulcers; α-pinene being the main agent responsible for the *Helicobacter pylori* antibacterial activity [21]. In a study about several EOs (*C. alba* was not included) and their effects on *H. pylori*, eugenol was detected in the EOs isolated from *Cinnamomum zeylanicum* and *Eugenia caryophyllus* [22]. Eucalyptol was also found in *Satureja montana* and also in *Eucalyptus globulus*. In addition to this, carvacrol (an α-terpineol isomer), was present in *C. zeylanicum* and *S.montana*. Little is known regarding the mechanisms of action of these compounds. For instance, carvacrol, has a hydroxyl group with the potential capability to act as a metal chelator of transmembrane carriers for monovalent cations thus affecting membrane potential [23]. Therefore, this compound potentially destroys the proton motive force and inhibits some enzymes and other essential macromolecules involved in this phenomenon [24]. With the above-described, one could associate the monoterpene α-terpineol and eucalyptol with the CA_EO antibacterial activity. With this background, the antibacterial activity could be attributed to CA_EO monoterpenes such as α-terpineol and eucalyptol. 

Regarding the lower MIC or bacteriolytic activity values reported against *H. pylori* for some EOs such as 0.01% (*v*/*v*) dilution for lemongrass (*Cymbopagon citratus*) oil [25] and the MIC value 2 µg/mL of EO from the Peruvian native plant *Minthostachys mollis* [26]. The MCB values for carrot and lemongrass oil are 0.02 and 0.04 µg/mL respectively [22]. These numbers are not quite comparable given that the value described here for *C. alba* EO 0.1%(*v*/*v*) dilution and MIC = 29 µg/mL, (Table 3) is close to the range of those oils previously mentioned as values required to block *H. pylori* growth. This implies that CA_EO is a promising candidate to fight *H. pylori* infections. For further comparison with other compounds also see Table 3. In addition to this antibacterial activity, the CA_EO has been used in, traditional medicine and an insect repellent. Other studies have verified antifungal properties against *Penicillium* sp. and *Fusarium oxysporum* [9] as well as activity against other fungus species [27,28].

In order to evaluate the selective cytotoxicity and the antitumoral activity of the EOs, mammary tumor cell line MCF-7 and the non-tumor cell line MCF10A, were incubated with CA_EO and LS_EO at different concentrations and a proliferation CV assay was performed [29]. CA_EO and LS_EO significantly inhibited the proliferation of mammary tumor MCF-7 cells at 64 and 32 ug/mL concentrations, being more evident with CA_EO. Conversely, in the MCF10A non-tumoral cells no significant differences were found among the treatments, irrespective of the concentration (Figure 2). We found that the MCF-7 epithelial cells showed an inhibited proliferation when treated with CA_EO at different concentrations (64 to 16 ug/mL). Minor concentrations of 8 ug/mL did not show any effects (Supplementary Figure S3). The antitumoral effect was maintained in the other tumor epithelial cells. The human epithelial renal cells (HK-2, 786-O and ACHN) were exposed to the EOs and a dose-response curve was performed. In contrast to the effect observed in non-tumoral mammary cells, the CA_EO showed an inhibitory effect on proliferation of non-tumor renal cell HK-2 (Figure 3a), while LS_ EO did not show an inhibitory effect on the proliferation of this cell line (Figure 3d). Regarding the primary tumor (786-O) and metastatic site (ACHN) renal cell lines, treatment with both EOs demonstrated a significant inhibition on cell viability and proliferation (Figure 3b–c and Figure 3d–e respectively). The CA and LS EOs demonstrated a significant inhibition on cell viability and proliferation at 64 ug/mL in human glioblastoma cells tumor cell line U87MG and human fibroblast cells (Figure 4 and Figure 5, respectively). The LS_ EO showed a greater inhibitory effect on these cell lines compared to CA_EO. This could be attributed to the presence of limonene which has been previously described for its antitumoral potential, inhibited the growth of lung cancer cells and suppressed the growth of transplanted tumors in nude mice [30]. Limonene exerted its effects by up-regulation of BAX, cytochrome c release, caspase-3, caspase-9, TGF-β, and down-regulation of anti-apoptotic Bcl-2 [31]. On the other hand, POH also up-regulates Bak, caspase-3, FasL, TGF-β, c-fos, and c-Jun as well as blocks extracellular signal-regulated kinase (ERK)-1/2 phosphorylation pathway [31,32]. Furthermore, both limonene and POH could possibly inhibit tumor progression through down-regulation of basal production of vascular endothelial growth factor (VEGF) in cancer cells [16]. Additionally, they also suppressed the mevalonate pathway as well as isoprenylation of small G proteins leading to tumor regression [33,34].

Plants are hence a potential source of drug discovery and development of cancer chemoprevention or treatment. It has been reported that they produce secondary metabolites with antitumoral effect available for the treatment of cancer patients [35]. We found antitumoral activity with a selective cytotoxicity of human tumor epithelial cells lines such as MCF-7 (mammary) and 786-O and ACHN (renal). The viability and proliferation were significantly affected by the treatment. Meanwhile, the EOs did not show any cytotoxic effect on non-tumor human epithelial cell lines MCF10A (mammary) and HK2 (renal). More studies, however, are necessary to carry out the anti-cancer activity of EOs and also their source of biological and chemical diversity [36]. The unique and complex structures of natural products cannot be obtained easily by chemical synthesis. Interest in medicinal plant research has increased in recent years, especially for the treatment of cancer [37]. Cytotoxicity has been reported for many EOs [34]. Despite the new evidence that has been reported, very few studies have been carried out on the combination of EOs with chemotherapy drugs, (e.g., the association synergistic effect of Geraniol and 5-fluorouracil tested in both SW620 and Caco-2 human colon carcinoma cell lines [32,38].

The CA_ EO showed low or no toxicity against *C. elegans* (Figure 6) but the LS_ EO showed toxicity in all the concentrations evaluated with 100% mortality both at 24 and 48 h. The CA_EO was toxic at 24 h at 50% with 100% mortality at 48 h. At 6.25 mg/mL, however, it showed toxicity at 48 h with 55% mortality. Although some level of toxicity was observed, it was only at the highest concentrations, higher than those recommended for studies in *C. elegans* (1 mg/mL) of the authors [19]. This demonstrates that CA_EO has low or no toxicity as opposed to the high toxicity of LS_EO.

The CA_EO showed some level of toxicity at the highest concentrations (50–3.125 mg/mL) higher than those recommended for studies in *C. elegans* (1 mg/mL), Forno [39], demonstrating that this oil presented low or no toxicity. Similar to what was reported Larrazabal [19], using oils from *Rica rica* and *Copa copa* showed low toxicity in *C. elegans*. Other studies reported marginal toxicity of EO obtained from *Amomum subulatum* [40]. On the other hand, tests with LS_EO showed high toxicity in all concentrations used against *C. elegans* (50–0.39 mg/mL). Similarly, Enam [41] reported nematocidal activity against *C. elegans* of *thymol* and *p-cymene*, ratified by Isman [42], reported a high toxicity effect of *p-cymene*.

The limitation of this study was that the biological activity of the major monoterpenes was not determined, and it would be an interesting topic to address in future studies.

## 4. Materials and Methods

### 4.1. Composition 

Gas chromatography–mass spectrometry (GC-MS) analysis was performed on a Varian gas chromatograph series 431 (Agilent Technologies, Inc., Santa Clara, CA, USA) fitted with a DB-5 ms fused silica capillary column (30 × 0.25 mm; film thickness, 0.25 μm) using split/split-less injection and coupled to a series 220 mass detector (Agilent Technologies, Inc.). The following conditions were used: injection volume: 0.8 μL with split ratio 1:80; helium as a carrier gas at 1.5 mL/min in constant flow; injector temperature: 250 °C; oven temperature: 50–260 °C at 2 °C/min. The mass spectra electron impact (EI+) mode was set at 70 eV, with an ion source temperature of 260 °C. The mass spectra were recorded within a range of 40–300 atomic mass units. Identification of the EO constituents was accomplished based on the following: the retention index (RI) determined with respect to a homologous series of *n*-alkanes (C_5_–C_28_; PolyScience, Niles, IL, USA) under the same experimental conditions; co-injection with standards (Sigma-Aldrich) and standard isolates; identification using an MS library (NIST 05 and Wiley; NIST/EPA/NIH Mass Spectral Library with Search Program (data version NIST 11; software version 2.0 g), available online at: http://www.nist.gov/srd/nist1a.cfm) and comparison with previously reported MS data [43].

### 4.2. Collection Identification and Extraction

The aerial parts of *Cryptocarya alba* (Molina) Looser (synonym *Peumus alba* Molina, vernacular name peumo), Lauraceae were collected at the beginning of the flowering season in September 2017 from Altos de Chicauma (33°00′–33°17′ S and 70°53′–71° 00′ W), Santiago Province, in the central region of Chile and *Laurelia sempervirens Tul.* (synonym *Atherosperma sempervirens*) were collected at the beginning of the flowering season in September 2017 from Curiñanco (39°48′30″ S and 73°14′30″ W) Valdivia, Chile.The plant was identified by Dr. Ecotoxicology Carlos Valdovinos and a voucher specimen was deposited at the Herbarium of the Facultad de Ciencias Químicas y Farmacéuticas, Universidad de Chile (CA-N_O_. 22,472 SQF, LS-N_O_. 22776). The EO was extracted by water distillation in a Clevenger-type apparatus. The EO was dried over and stored as previously described [40] until further analysis. 

### 4.3. Antioxidant Capacity

The total polyphenol content (TPC) and the antioxidant capacity of the oils were determined by the ferric reducing antioxidant power (FRAP) and 1,1-diphenyl-2-picrylhydrazyl (DPPH) assays using Trolox (53188-07-1, Sigma-Aldrich, St. Louis, MO, USA) as a standard, according to the protocols described by [44] and adapted for Synergy™ HTX multimodal microplate reader, equipped with dual reagent injector module (BioTek Instruments, Inc., Winooski, VT, USA). All assays were performed in Corning^®^ Costar^®^ 96-well microplates (Corning Life Sciences., Oneonta, NY, USA).

#### 4.3.1. Chemical Materials

1,1-diphenyl-2-picrylhydrazyl radical (DPPH), 2,2’-azino-bis(3-ethylbenzothiazoline-6-sulfonic acid) diammonium salt (ABTS), Folin & Ciocalteu’s phenol reagent, 2,4,6-*tris*-(2-pyridyl)-s-triazine (TPTZ), (±)-6-hydroxy-2,5,7,8-tetramethylchromane-2-carboxylic acid (Trolox), potassium persulfate (K_2_S_2_O_8_), and gallic acid were purchased from Sigma-Aldrich (St. Louis, MO, USA). Sodium carbonate (Na_2_CO_3_), iron (III) chloride hexahydrate (FeCl_3_ 6H_2_O), hydrochloric acid (HCl), methanol (MeOH), sodium phosphate monobasic (NaH_2_PO_4_) and sodium phosphate dibasic (Na_2_HPO_4_) were purchased from Merck (Darmstadt, Germany).

#### 4.3.2. Total Polyphenol Content (TPC) Estimation

The TPC method was based on the 96-well microplate Folin–Ciocalteu method and adapted from [12]. A 20 μL sample of the diluted EO (500 μg/mL) was mixed with 100 μL of 10% (*v*/*v*) Folin-Ciocalteu reagent and shaken. The mixture was left for 5 min, and then 80 μL of sodium carbonate solution (700 mM) was added, and the mixture was shaken for 1 min. After 60 min at room temperature, the absorbance was measured at 765 nm using a microplate reader. Gallic acid dilutions (0–1000 μg/mL) were used as standards for calibration. The results were expressed as mg of gallic acid equivalent per ml of essential oil. All experiments were performed in triplicate. 

#### 4.3.3. FRAP Assay

The FRAP assay was carried out with the following method [44]. The FRAP reagent consisted of 300 mM acetate buffer pH 3.6, 10 mM 2,4,6-*tris*-(2-pyridyl)-s-triazine (TPTZ) solution in 40 mM HCl, and 20 mM FeCl_3_·6H_2_O aqueous solution at a ratio of 10:1:1 (*v*/*v*). The EOs were prepared at a final concentration of 500 μg/mL. The extract solution (10 μL) was mixed with 70 μL of freshly prepared FRAP solution and incubated at 37 °C for 30 min. The absorbance of the solutions was measured at 593 nm. Trolox was used as the standard solution to construct a calibration curve over a concentration range of 0–500 μg/mL. The FRAP results were expressed as mg of Trolox equivalent per mL of EO. All experiments were performed in triplicate.

#### 4.3.4. DPPH Radical Scavenging Activity Assay

The quantitative measurement of the EOs radical scavenging properties was carried out by the following methodology [16]. A 0.2 mM solution of DPPH in methanol was prepared, and 70 μL of this solution was added to 20 μL of EO (0–1000 μg/mL). Trolox at concentrations of 0–1000 μg/mL was used as a reference antioxidant. Discoloration of reaction mixture was measured at 517 nm after incubation for 30 min. The results were expressed as IC_50_ values (concentration of EO in μg/mL required to inhibit 50% of DPPH radical present in solution). The analyses were carried out in triplicate.

#### 4.3.5. ABTS Radical-Scavenging Activity Assay

The radical scavenging capacity of the samples for ABTS radical cation was carried out by following method. ABTS was generated by mixing a 7 mM stock solution of ABTS in PBS at pH 7.4 with 2.5 mM K_2_S_2_O_8_ (final concentration) followed by storage in the dark at room temperature for 16 h before use. The mixture was diluted with PBS to give an absorbance of 0.700 ± 0.02 units at 734 nm using spectrophotometer. For each sample, diluted solution of the sample (20 μL) was allowed to react with fresh ABTS solution (180 μL), and then the absorbance was measured 6 min after initial mixing. The blank was prepared from 180 μL of solution of the ABTS in PBS and 20 μL of PBS. Trolox was used as a standard and the capacity of free radical scavenging was expressed by IC_50_ (μg/mL) values calculated denote the concentration required to scavenge 50% of ABTS radicals. All measurements were performed in triplicate [45,46].

### 4.4. Antibacterial Activity 

#### 4.4.1. Chemical Materials and Antibiotics 

α-Terpineol, β-phellandrene, and eucalyptol were purchased from Sigma Aldrich (99% pure), while the antibiotic cocktail DENT^®^ (SR014E), which is selective for *H. pylori* (HP) growth, was purchased from Oxoid, USA and contained 10 mg/mL vancomycin; 5 mg/mL cefsulodin; 5 mg/mL amphotericin B and 5 mg/mL trimethoprim. Other antibiotic stocks used were 100 mg/mL ampicillin and 12.5 mg/mL tetracycline, both from Sigma Aldrich (St. Louis, MO, USA).

#### 4.4.2. Microbial Strains

For the microbiological studies the bacteria strains used were: 16 *S. aureus* (15 clinical isolates and the reference strain ATCC 25923), 16 *E. coli* (15 clinical isolates and the reference strain ATCC 25922) were obtained from the collection of Laboratorio de Microbiología, Departamento de Tecnología Médica, Universidad Diego Portales kindly provided by Pedro Cortés and 15 *C. albicans* and *C. parapsilosis* (ATCC 22019) (a reference strain used in genus *Candida* assays, kindly provided by Dr. Alvarez, Instituto de Ciencias Biomédicas, Facultad de Medicina, Universidad de Chile. The strains were grown in Mueller Hinton Agar and incubated at 37 °C. 

Seven *H. pylori* (HP) strains were selected from a collection of clinical isolates from the Laboratorio Patogenesis Microbiana, Facultad de Medicina, Universidad Diego Portales, Santiago, Chile. The HP strains were routinely cultured on 1.5% Brucella agar (from BD, USA) supplemented with 5–7% horse blood and DENT cocktail diluted 1:200 and were incubated for 3–7 days under microaerophilic conditions (10% CO_2_) and 95% humidity at 37 °C. The *HP* strains were confirmed by colony morphology, Gram staining, the urease test (He-Py Test, GrupoBios, Chile) and positive PCR using primers for the *H. pylori*-specific *hpaA* gene, which has been previously described (Bergozelli et al., 2003). *HP* strain HPK5 was used as a pathogen reference [47,48].

#### 4.4.3. Agar Disk Diffusion Assay

The agar disk diffusion technique has been widely used to assay plant extracts for antimicrobial activity. In this method, 6 mm sterilized filter paper disks (Whatman^®^ No 1) were saturated with 10 μL of filter-sterilized oil plant extract. The impregnated discs were then placed onto the surface of a suitable solid agar medium, such as Mueller Hinton BD^®^. The media was pre-inoculated with test organisms. A standard inoculum size of 1 × 10^8^ CFU/mL of bacteria was used to inoculate the diffusion plates, which is equal to the McFarland 0.5 turbidity standard. The plates were incubated overnight at 37 °C, and the diameter of the inhibition zone around each disk (diameter of inhibition zone plus diameter of the disk) was measured in millimeters. A Sensydisc was used as a positive control for Gram+ and Gram− strains Sigma Aldrich (St. Louis, MO, USA) [48].

#### 4.4.4. Microplate Assay 

The microdilution method was performed in ELISA plates according to instructions approved by the Clinical and Laboratory Standards Institute. For this purpose, the oil was added in 10-fold decreasing serial concentrations into Mueller Hinton broth BD^®^. Fresh bacterial suspensions (equivalent to 1 × 10^8^ bacteria/mL) were used to inoculate the microplates and were incubated as described above [48].

To enhance EO solubility, they were dissolved in 2% (*v*/*v*) dimethyl sulfoxide (DMSO) Merck^®^ (this amount does not affect bacterial growth) and then added to the microplates. The inoculated microplates were incubated as described above for agar plates, but for only 1 day. ELISA (Tecan) readings at 600 nm were recorded to calculate the MIC values as described by the Clinical and Laboratory Standards Institute [48,49] protocol. Control microplates of bacteria without the EO and without bacteria were also included in these assays. To identify significant differences between treated and untreated groups, data comparison by the ANOVA test was performed using the GraphPad Prism 5 program.

### 4.5. Cytotoxicity

#### 4.5.1. Cell Lines Culture

The human epithelial mammary cell lines MCF10A (non-tumoral) and MCF7 (tumoral) and the human epithelial renal cell lines HK-2 (non-tumoral), 786-O and ACHN (tumoral) were obtained from American Type Culture Collection (ATCC, Rockville, MD). Glioblastoma cell lines U87GM and Primary human fibroblasts. The cells were cultured in Dulbecco’s Modified Eagle’s F-12 Medium (DMEM-F12; Gibco, USA) supplemented with 10% fetal bovine serum (FBS; Gibco, USA) and maintained at 37 °C in 5% of CO_2_ atmosphere. When the cells reach the 80% of confluence were replicated. The numbers of generations of the cell lines were about 5–10. In vitro assays were performed in triplicate of three independent experiments for each cell line.

#### 4.5.2. Chemical Materials

Trypsin-EDTA 0.5% (Gibco, USA), PBS 1X (Gibco, USA), DMSO, crystal violet, ethanol, Na_2_HPO_4_, DMEM F12, FBS, MTT, HCl, were purchased from Sigma-Aldrich (St. Louis, MO, USA).

#### 4.5.3. Crystal Violet Proliferation Assay

To evaluate the dose-response at given concentrations and the time-course of Eos, the cells where detached with Trypsin-EDTA 0.5% (Gibco, USA) and 5 × 10^3^ cells/well were seeded and incubated in a 96-well cell plate with DMEM-F12 supplemented with 10% FBS for 24 h. The supernatant were removed and the cells were washed 1× with PBS 1X (Gibco, USA), and treated with EOs extracts of CA or LS at known concentrations (64, 32, 16, 8, 4, or 2 µg/mL) dissolved in culture medium (DMEM-F12 supplemented with 1% FBS). The vehicle was DMSO 1% dissolved in DMEM-F12, supplemented with 1% FBS as a control treatment (the EOs are dissolved in DMSO 1%). After 24 or 48 h the supernatant was discarded, the cells were washed with PBS 1X and incubated with 100 µl of crystal violet (CV) solution (0.2% *w*/*v* in ethanol 10%) (Gibco, USA) for 20 min, then the CV solution was removed and Na_2_HPO_4_ (0.1 M, pH 4.5, in ethanol 50:50 *v*/*v*) (SIGMA, USA), was added to elute the intra-cellular colorant. The absorbance of each sample was measured at 570 nm. The results are shown as percentage of color intensity and normalized to cells grown in DMSO 1% as control treatment. 

#### 4.5.4. MTT Proliferation Assay

To evaluate the dose-response at given concentrations of EOs, 5 × 10^3^ cells/well were seeded and incubated in a 96-well cell plate with DMEM-F12 10% FBS for 24 h. The cells were treated with EOs extracts at known concentrations (64, 32, 16 µg/mL) dissolved in culture medium (DMEM 1% FBS) or control medium (DMSO 1% in DMEM 1% FBS). After 24 or 48 h the supernatant was discarded and MTT solution was added to each well, according to the manufacturer’s recommendation. The cells were incubated in dark at 37 °C for 2 h. After that, the MTT solution was removed and acidified isopropanol (4% of HCl 1N) was added. The absorbance of each sample was measured at 570 nm. The results are shown as the percentage of color intensity and normalized to cells grown in DMSO 1% as control treatment.

#### 4.5.5. Statistical Analysis

The results were analyzed by ANOVA One way for independent data with Tukey’s test post-hoc. A *p* < 0.05 was considered as statistically significant.

### 4.6. Toxicity

#### 4.6.1. Chemical Materials 

NaCl, nematode growth medium, KH_2_PO4, Na_2_HPO4, MgSO4, DMSO, Ethanol absolute, were purchased from Sigma-Aldrich (St. Louis, MO, USA).

#### 4.6.2. Maintenance of Caenorhabditis Elegans Culture

The N2 wild-type strain of *C. elegans* was used to investigate toxicity. The nematodes were maintained on nematode growth medium (NGM) agar plates, with an established layer of the *E. coli* OP50 strain. The plates were maintained at 20 °C for 3 days. The gravid nematodes were collected and treated for 5 min in a chlorine solution (0.45 N NaOH, 2% HOCl Sigma Aldrich (St. Louis, MO, USA)) to isolate eggs. The eggs were placed in plates with OP50; once hatched they were left for 3 days to obtain synchronized adult nematodes. These were collected in M9 saline solution (1.5 g KH_2_PO_4_, 3 g Na_2_HPO_4_, 2.5 g NaCl, 0.5 mL of 1 M MgSO_4_ Sigma Aldrich (St. Louis, MO, USA) and distilled water for a final volume of 500 mL) [50].

#### 4.6.3. Test Preparation 

The CA and LS essential oils were prepared at concentrations of 0.39, 0.78, 1.56, 3.12, 6.25, 12.5, 25 and 50.0 mg/mL and placed in a final volume of 100 µL in 96-well plates. The control assays were performed with M9 and 1% DMSO Sigma Aldrich (St. Louis, MO, USA). Ten individuals of *C. elegans*/well were used in each trial. The plates were incubated (incubator Binder KT) at 20 °C for 24 and 48 h. The experiments were performed in triplicate and repeated twice. After incubation at 20 °C, all the nematodes were counted at 24 and 48 h to determine their survival. They were considered alive if they showed some type of motility and were considered dead when they showed no movement, either from their tail, head or pharynx, after 5 s of observation (Stereo Microscope Leica MZ12). The count was performed to establish the degree of mortality [51].

## 5. Conclusions

This study shows new interesting knowledge about EOs extracted using hydrodistillation in Clevenger apparatus *Cryptocarya alba* and *Laurelia sempervirens*. The chemical composition analyzed by GC-MS allowed us to determine the major components present in each species, which could be responsible for the biological activity they present, in future studies the activity of each one of them could be studied. Both EOs showed antimicrobial activity in different concentrations to different bacteria. Due to great antibacterial activity, along with the composition of EOs, we see a great potential for future usages of these oils, such as natural antimicrobials or food preservatives. Additional studies, including pharmacological, toxicological, and clinical aspects and formulation of these compounds, will be required to understand the role of some components of CA and LS_EOs to demonstrate the remarkable efficacy and safety of these EOs as antimicrobial agents. The CA and LS_EOs have important bioactive potential, cytotoxicity and toxicity, which would allow their use of active ingredients in fields such as the clinical, nutrition, cosmetics, sanitation and plaguicides, among others.

## Figures and Tables

**Table 1:** Compounds from GC-MS analyses of (A) *C. alba* and (B) *L. sempervirens* EOs, the respective calculated Kovats index (KI cal) and from the Kovats index literature (KI lit).

Fraction Number	Retention Time (min)	CAS	KI Cal	KI Lit	[M^+^]	Fragment	%	Name
(A) *C. Alba* Essential Oil								
1	14.5	99-83-2	-	1007	136	99 (100) 91 (38) 77 (33)	0.71	d-phellandrene
2	14.8	7785-70-8	1025	939	136	93 (100) 92 (41) 79 (19)	3.88	1*R*-α-pinene
3	15.7	79-92-5	1041	953	136	93 (100) 121 (66) 41 (38)	0.19	Camphene
4	17.0	555-10-2	1081	1035	136	93 (100) 43 (38) 121 (23)	14.84	β-phellandrene
5	17.3	18172-67-3	1012	981	136	93 (100) 41 (69) 69 (43)	4.18	l-β-pinene
6	19.5	586-62-9	1085	1088	136	93 (100) 121 (98) 136 (72)	0.92	Terpinolene
7	19.9	527-84-4	1067	1014	136	93 (100) 135 (75) 121 (46)	3.99	*o*-cimol
8	20.1	5989-54-8	1050	1031	136	68 (100) 93 (57) 39 (36)	3.41	Limonene
9	20.3	470-82-6	1013	1030	154	139 (100) 154 (85) 27 (28)	21.63	Eucalyptol
10	21.7	99-85-4	1035	1057	136	93 (100) 91 (35) 136 (33)	2.67	γ-terpinene
11	24.3	54410-94-5	1053	1116	170	68 (100) 57 (43) 41 (39)	1.34	3 methyl 3 butenyl
12	26.6	562-74-3	1079	1177	154	71 (100) 111 (53) 43 (45)	1.72	4-terpineol
13	27.2	98-55-8	1009	1189	154	59 (100) 93 (53) 43 (45)	24.96	α- terpineol
14	33.3	17699-05-7	1580	1434	204	93 (100) 119 (86) 41 (51)	2.88	α-bergamolene
15	34.8	339154-91-5	1575	1430	204	121 (100) 93 (69) 41 (63)	0.99	γ-elemene
16	35.3	483-77-2	1515	1523	202	132 (100) 159 (98) 131 (51)	1.36	calamenene
17	38.1	473-15-4	1733	1645	223	207 (100) 125 (19) 153 (15)	1.49	β-eudesmol
(B) *L. Sempervirens* Essential Oil								
1	12.0	138-86-3	-	1036	136	93 (100) 68 (67) 136 (63)	5.3	Limonene
2	12.8	13877-91-3	1000	1023	136	93 (100) 91 (57) 41 (54)	1.3	*O*-cimene
3	20.3	120-58-1	1000	-	162	162 (100) 104 (31) 78 (13)	91.9	Isozafrol
4	24.7	489-39-4	1148	-	204	41 (100) 161 (92) 91 (86)	0.5	Aromadendrene
5	25.2	23986-74-5	1500	1499	204	161 (100) 105 (79) 41 (62)	0.7	Germacrene D
6	25.6	339154-91-5	-	1433	204	121 (100) 93 (69) 41 (63)	0.3	γ-elemene

NIST 2018.

**Table 2:** Antioxidant capacity of *C. alba* and *L. sempervirens* essential oils.

Essential Oils	Total Phenols ^a^	FRAP ^b^	IC_50_ DPPH ^c^	IC_50_ ABTS ^c^
*L. sempervirens*	63.7 ± 10.7	229.8 ± 11.1 *	417.8 ± 5.8 ^+++^	401.2 ± 8.7 ^+++^
*C. alba*	163.6 ± 10.7 **	166.8 ± 27.9	492.7 ± 11.1 ^+++^	203.0 ± 12.8 **^,+++^
Trolox	--	--	11.7 ± 2.1	35.6 ± 1.5

All values were expressed as Means ± SEM (*n* = 4). **^a^** expressed in mg Gallic acid equivalent/g essential oil. **^b^** expressed in mg Trolox equivalent/g essential oil. **^c^** expressed in µg/mL. *^,^ ** significantly different (*p* < 0.5 and *p* < 0.01). ^+++^ significantly different respect to control (*p* < 0.001).

**Table 3:** The MIC values of CA_EO, LS_EO and their major components against clinical isolates of *H. pylori, S. aureus*, *E. coli* and *C. albicans.*

EO or Its Purified Component	MIC (μg/mL) *H. pylori*	MIC (μg/mL) *S. aureus*	MIC (μg/mL) *E. coli*	MIC (μg/mL) *C. albicans*
CA_EO	30	19	36	31
α-terpineol	27	32	16	16
β-phellandrene	30	32	32	32
Eucalyptol	30	32	32	32
LS_EO	64	64	64	64
Limonene	32	32	64	64

The ANOVA analysis including the Tukey test did not show significant differences between the β-phellandrene and eucalyptol purified compounds. The values were an average of 7 isolates for *H. pylori* and 15 isolates for the other species. The whole experiment was done in triplicate.

## Supplementary Materials

The following are available online, Figure S1: Chromatographic GC profile of *L. sempervirenes* essential oil, Figure S2: Chromatographic GC profile of *C. alba* essential oil, Figure S3: Dose-response curve of human epithelial mammary tumor cell line MCF-7 treated with CA (peumo) and LS (laurel) EOs.

## Abbreviations

EOs: Essential Oils; MC: majority compounds; TPC: Total polyphenol content; AC: Antioxidant capacity; GC–MS: Gas chromatography–mass spectrometry; MIC: Minimum inhibitory concentration; HD: hydrodistillation; CA: *Cryptocarya alba* (Peumo); LS: *Laurelia sempervirens* (Laurel).
