# Supplementary material for: The Chemical Compositions of Essential Oils Derived from Cryptocarya alba and Laurelia sempervirens Possess Antioxidant, Antibacterial and Antitumoral Activity Potential
Source: Molecules. 2020 Nov 28;25(23):5600. doi: 10.3390/molecules25235600 (PMC7729746; doi:10.3390/molecules25235600)
Supplement: Supplementary file 1 [file molecules-25-05600-s001.pdf]

SUPPLEMENTARY DATA

The chemical compositions of essential oils derived from *Cryptocarya alba* and *Laurelia sempervirens* possess antioxidant, antibacterial and antitumoral activity potential.

Jorge Touma[a], Myriam Navarro[a], Betsabet Sepúlveda[b], Alequis Pavon[c], Gino Corsini[c], Katia Fernández[a], Claudia Quezada[e], Angelo Torres[e], María José Larrazabal-Fuentes[d], Adrian Paredes[d], Ivan Neira[d], Matías Ferrando[f], Flavia Bruna[f], Alejandro Venegas[a] and Jessica Bravo[a,*]

[a] Facultad de Medicina, Centro de Investigación Biomédica, Universidad Diego Portales, Ejército 141, Santiago – Chile

[b] Facultad de Ciencias Químicas y Farmacéuticas, Universidad de Chile, Santos Dumont 964, Santiago-Chile

[c] Instituto de Ciencias Biomédicas, Facultad de Ciencias de la Salud, Universidad Autónoma, Santiago Chile

[d] Universidad de Antofagasta, Departamento de Ciencias de los Alimentos y Nutrición, Departamento de Tecnología Médica FACSA, Laboratorio Química Biológica, Angamos 601, Antofagasta, Chile.

[e] Universidad Austral, Laboratorio de Patología Molecular, Instituto de Bioquímica y Microbiología, Facultad de Ciencias, Universidad Austral de Chile, Valdivia, Chile

[f] Instituto de Medicina y Biología Experimental de Cuyo (IMBECU), Laboratorio de Hormonas y Biología del Cáncer, CONICET CCT-Mendoza UNcuyo, Argentina

***** Correspondence: Corresponding author: Jessica Bravo. Laboratorio de Productos Bioactivos, Facultad de Medicina, CIB, Universidad Diego Portales, Ejército 141, código postal 8370007, Santiago – Chile. Tel: +562 26762934; Email address: jessica.bravo@udp.cl

Received: date; Accepted: date; Published: date

Abstract: *Cryptocarya alba* (Peumo; CA) and *Laurelia sempervirens* (Laurel; LS) are herbs native to the Chilean highlands and have historically been used for medicinal purposes by the Huilliches people. In this work, the essential oils were extracted using hydrodistillation in Clevenger apparatus and analyzed by GC-MS to determine their composition. The antioxidant capacity (AC) were evaluated *in vitro*. The cytotoxicity was determined using cell line cultures both non tumoral and tumoral. The toxicity was determined using the nematode *Caenorhabditis elegans*. The antimicrobial activity was evaluated against 52 bacteria using the agar disc diffusion method and the minimum inhibitory concentrations (MICs) were determined. The principal compounds found in *C. alba* essential oil (CA_EO) were α-terpineol (24.96%) and eucalyptol (21.63%) and were isazafrol (91.9%) in *L. sempervirens* essential oil (LS_EO). Both EO showed antioxidant capacity *in vitro*. Both EO showed antibacterial activity against bacteria using. LS_EO showed more inhibitory effect on these cell lines respect to CA_EO. Both EO showed toxicity against the nematode *C. elegans* at 3.12- 50mg/mL. The essential oils of CA and LS have an important bioactive potential in their antioxidant, antibacterial and cytotoxicity activity. Both essential oils could possibly be used in the field of natural medicine, natural food preservation, cosmetics, sanitation and plaguicides among others.

Keywords: Essential oil; *Cryptocarya alba*, *Laurelia sempervirens*, Chemical composition; Antioxidant; Cytoxicity; Toxicity; Antimicrobial.

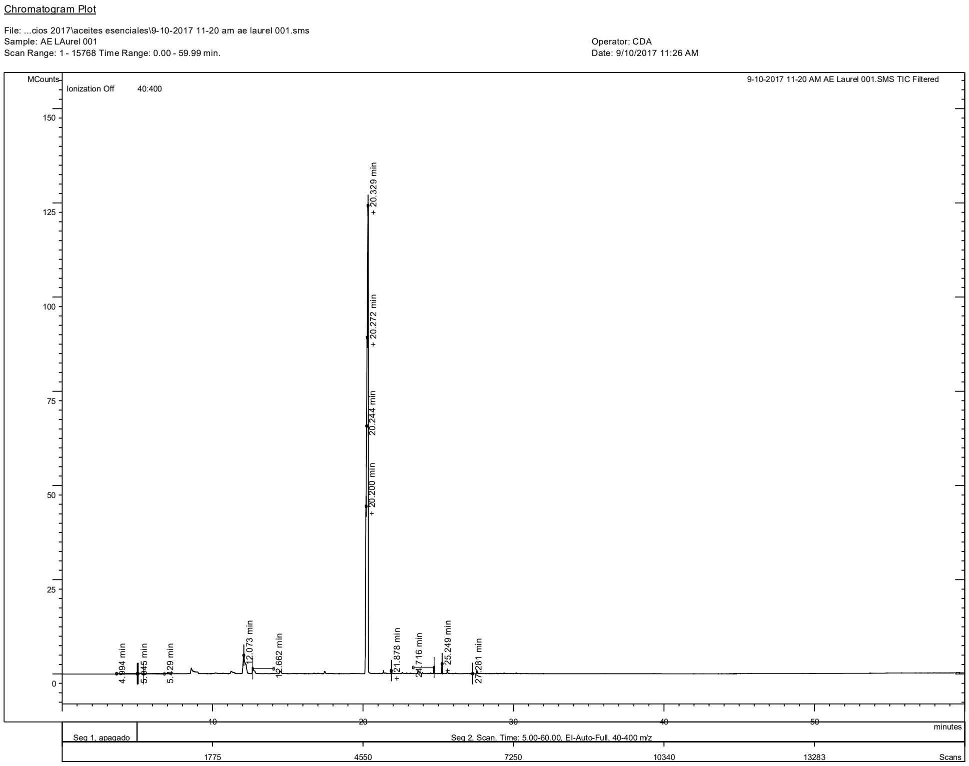

Figure S1. Chromatographic GC profile of *L. sempervirenes* essential oil.

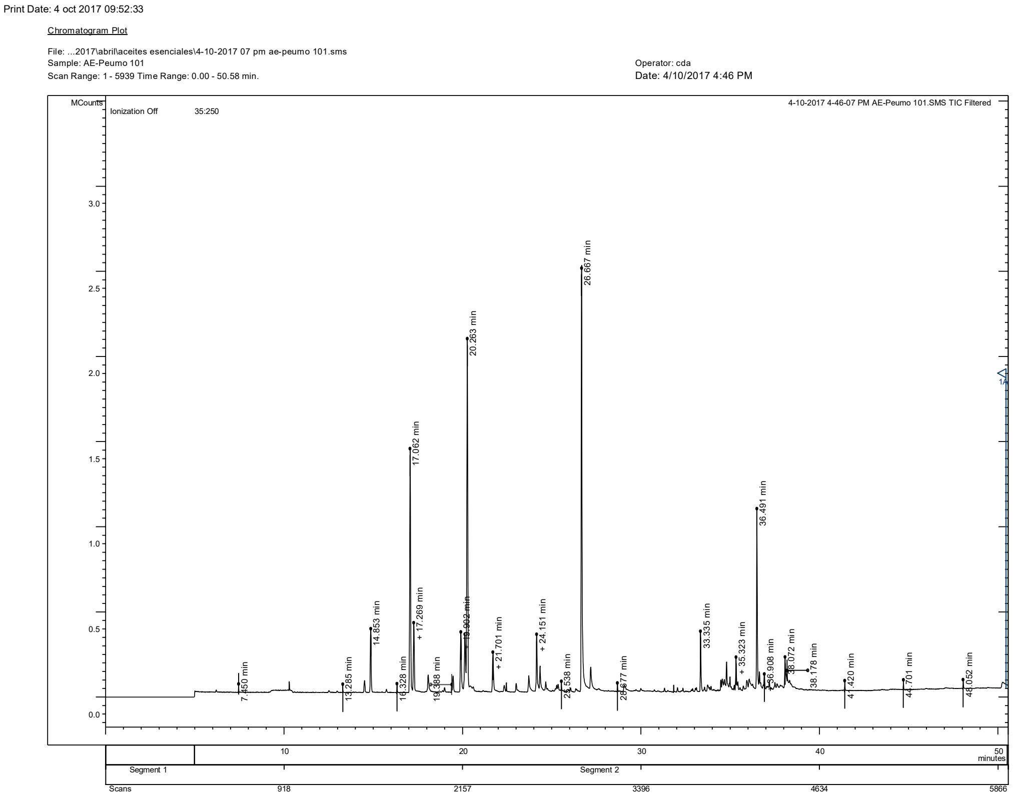

Figure S2. Chromatographic GC profile of *C. alba* essential oil.

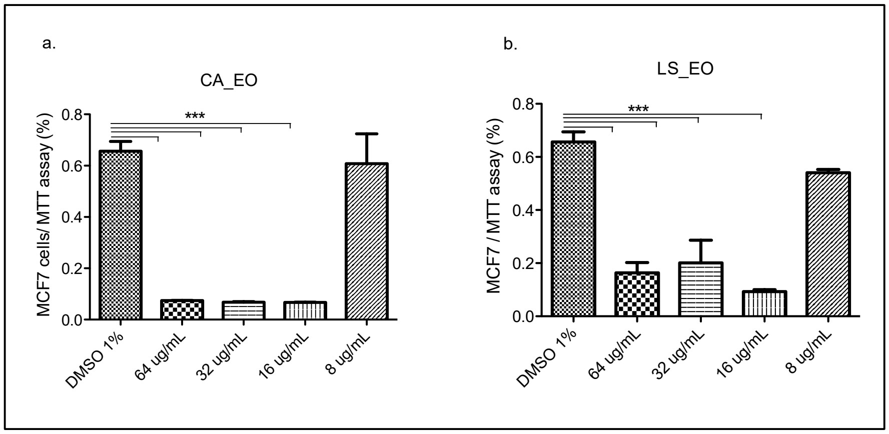

Figure S3. Dose-response curve of human epithelial mammary tumor cell line MCF-7 treated with CA (peumo) and LS (laurel) EOs. The graph bar corresponds to the proliferation of MCF-7 treated with the EOs (a. Peumo) and (b. Laurel), at different concentrations versus control (DMSO 1%) for 48 h, evaluated by MTT assay at 570 nm. Three independent trials were performed, in triplicate, for each treatment and concentration. ***$p < 0.001$.
